# Engaging in imagery versus verbal processing of worry: Impact on negative intrusions in high worriers

**DOI:** 10.1016/j.brat.2009.12.011

**Published:** 2010-05

**Authors:** Caroline Stokes, Colette R. Hirsch

**Affiliations:** a Institute of Psychiatry, King's College London, De Crespigny Park, London SE5 8AF, UK; b University of Western Australia, 35 Stirling Highway, Crawley WA 6009, Perth, Australia

**Keywords:** Generalised anxiety disorder, Worry, Verbal processing, Imagery, Negative thoughts

## Abstract

Chronic, excessive, and uncontrollable worry is the defining characteristic of generalised anxiety disorder. Worry largely consists of verbal thought and it has been postulated that this predominance of verbal thought in worry may contribute to its perseveration. In an investigation of this issue, high worriers were trained to engage in either imagery or verbal processing. Mentation was sampled before and after a five-minute period of worry during which participants engaged in either imagery of the worry topic or verbal processing of the worry topic. Verbal worry resulted in a significant increase in negative intrusions, consistent with previous research. Furthermore, imagery was associated with a decrease in negative intrusions. The results support the theory that the predominantly verbal nature of worry may be responsible for the uncontrollability and maintenance of worry.

## Introduction

Worry is a primary characteristic of anxiety, and has been described as a cognitive process “concerned with future events where there is uncertainty about the outcome, [but] the future being thought about is a negative one” (Dugas, 2004, p.5). Individuals with high levels of worry, both with and without a diagnosis of generalised anxiety disorder (GAD), experience worry as persistent, pervasive and uncontrollable. The extent of uncontrollability of worry is one feature that distinguishes high worriers and individuals with GAD from those without excessive worry (Borkovec, Robinson, Pruzinsky, & DePree, 1983). For example, high worriers instructed to worry have a greater number of subsequent negative intrusions during an attention task than non-worriers (Borkovec et al., 1983; Pruzinsky & Borkovec, 1990; York, Borkovec, Vasey, & Stern, 1987).

The question remains of why individuals prone to worry find it so difficult to disengage from their worry once it begins. Although anticipation of probable danger is adaptive, it is unclear why excessive worry persists when it often causes distress and has few apparent benefits. Many studies have demonstrated that the phenomenology of worry consists largely of verbal thought. For example, Borkovec and Inz (1990) demonstrated that both individuals with GAD and non-worriers reported a predominance of verbal thought during a period of worry, compared to a period of relaxation in which participants with GAD reported near equal amounts of imagery and verbal thought, and non-worriers reported a predominance of imagery. These findings were later replicated by East and Watts (1994) and extended by Behar, Zuellig, and Borkovec (2005) who compared a period of worry with a trauma recall condition. Again, they found that worry is experienced primarily as verbal thought, in contrast to trauma recall which is experienced primarily in images.

The cognitive avoidance hypothesis (Borkovec, Alcaine, & Behar, 2004; Sibrava & Borkovec, 2006) suggests that the predominantly verbal nature of worry functions as a means of avoiding potentially distressing imagery. In keeping with this, Butler, Wells, and Dewick (1995) showed participants a distressing video (of an accident at work) and then instructed them to either worry about it or to generate mental images from the video. While verbal worry was associated with a greater decrease in anxiety immediately after worrying about the video, it was also associated with more frequent intrusions of the video over the following three days compared to participants asked to generate imagery. This could suggest that verbal worry may be reinforced by initially reducing anxiety, but at the cost of preventing any longer-term reduction in anxiety and negative intrusions, perhaps due to lack of habituation or corrective learning about the topic, in line with Foa and Kozak's (1986) emotional processing theory.

Butler et al. (1995) investigated the differential impact of imagery and verbal processing on subsequent intrusions following a distressing video, but the question remains whether imagery about a personally-relevant current worry topic would lead to less subsequent negative intrusions than worry in verbal form. The present study was thus designed to compare the effects of worrying in verbal form or imagery on subsequent negative intrusions in volunteers reporting excessive worry. It was expected that worrying in verbal form would lead to more frequent subsequent negative intrusions than would worrying in the form of images.

Butler et al. (1995) and Nelson and Harvey (2002) demonstrated that generating imagery about a novel potentially worrisome topic (in the latter study, giving a speech) was associated with greater distress than verbally worrying about it. In keeping with this, it may be that participants generating imagery about their own worry topic would display a greater increase in negative affect over the worry period compared to those engaging in verbal worry. Exploring the effect of generating imagery about a personally-relevant worry topic on mood, compared to verbally worrying, was a secondary aim of the study.

## Method

### Design

High worriers were randomly allocated to engage in worry about a current worry topic in verbal form or imagery. The number of negative intrusions was assessed during a preceding baseline and post-worry breathing focus phase. Rating scales were administered throughout the experiment to assess mood.

### Participants

Participants were recruited from staff and students at King's College London. To ensure that all participants were high worriers, the Penn State Worry Questionnaire (PSWQ; Meyer, Miller, Metzger, & Borkovec, 1990) was administered. To be eligible, participants had to score 56 or above when initially recruited and on the day of testing^2^ in addition to completing the task correctly. The final sample consisted of 30 participants randomized to the imagery group (3 male) and 30 participants randomized to the verbal group (4 male). Participant characteristics and *t*-tests are shown in Table 1. Groups did not differ in age or scores on any of the questionnaire measures completed at baseline.

### Measures

Trait worry was measured using the PSWQ (Meyer et al., 1990). Trait anxiety and depressed mood were assessed using the following standardised self-report measures: Spielberger State-Trait Anxiety Inventory (STAI Form Y; Spielberger, Gorsuch, Lushene, Vagg, & Jacobs, 1983), the Hospital Anxiety and Depression Questionnaire (HADS; Zigmond & Snaith, 1983), and the Generalised Anxiety Disorder Questionnaire (GAD-Q-IV; Newman et al., 2002). Analogue scoring criteria for the GAD-Q-IV indicated that 18 participants in each group (verbal and imagery) were likely to meet criteria for GAD.

### Procedure

Participants first completed the PSWQ, the GAD-Q-IV, the STAI (state and trait versions), and the HADS. Participants then completed the worry task (see below for further details). Finally, they gave the experimenter a full account of any cognitive intrusions experienced during the worry task. After the experiment, participants were debriefed about the aims of the study and paid £12 ($18) for their time.

### Worry task

This task was adapted from an established experimental paradigm for assessing the impact of worry on subsequent thought intrusions (previously referred to as the breathing focus task; Borkovec et al., 1983; Pruzinsky & Borkovec, 1990; Ruscio & Borkovec, 2004; Hirsch, Hayes, & Mathews, 2009). It consisted of four stages: a five-minute baseline breathing focus phase; training in the designated type of mentation; a five-minute period of worry whilst engaging in imagery or verbal processing (depending on group allocation); and a five-minute post-worry breathing focus phase.

### Baseline breathing focus phase

Participants were instructed to focus their attention on their breathing. At random intervals a computer generated a tone to signal participants to report out loud whether their attention was indeed focused on their breathing, or whether at that moment they were experiencing a cognitive intrusion. If they had an intrusion, they rated it as positive, neutral, or negative, and gave a brief description. A total of 12 tones sounded during each breathing focus phase, and the researcher made a note of all ratings and descriptions given. At the end of the breathing focus phase, participants completed mood rating scales. These comprised of three visual analogue scales assessing degree of anxiety, depression, and happiness. Each scale measured 100 mm in length with anchors “not at all” at one end and “extremely” at the other. Participants indicated their mood during the breathing focus phase by marking a cross (*x*) on each scale.

### Training phase

After the baseline breathing focus phase, participants were trained in the designated style of mentation. To train participants to engage in imagery, imagery was first defined as: “generating an image of the situation and tuning in to what you can see, feel, smell, hear and taste in the image as though you are actually there right now”. Then in keeping with Holmes and Mathews (2005), participants were helped to imagine cutting a lemon. They were then asked to imagine a specified topic (eating dinner) and to generate and hold an image about three further non-worry topics for a minute each. After each practice, participants gave information on the extent to which they had engaged in the designated mentation style. If necessary, they were given feedback on how to generate imagery in further detail.

The training phase for participants in the verbal processing group followed a similar procedure, except that participants were asked to think “in words, sentences and questions, as though you are talking to yourself”. Again, the experimenter gave the example of thinking about cutting a lemon in words and sentences. Participants were then asked to practice by thinking in verbal form about four abstract topics for a minute each: “friendship”, “enjoyment”, “literature”, and “experience”. These topics were chosen because they were positive or neutral and therefore unlikely to trigger worry, and because they were abstract enough to minimize the chances of spontaneously generating a lot of imagery.^3^ This training procedure was developed, refined and found to be effective during a piloting stage.

### Five-minute worry phase

After the training phase, participants were asked to identify the topic currently worrying them the most. They briefly discussed this topic with the experimenter to ensure that it was related to a potentially negative future event, and the experimenter wrote a brief summary of the worry topic. Participants were then asked to silently worry about their topic using the designated type of mentation (verbal processing or imagery) for five minutes, during which time the researcher left the room. At the end of the five minutes, the experimenter returned.

### Post-worry breathing focus phase

Immediately following the worry phase, a second breathing focus phase and associated mood ratings were completed. This followed the same procedure, and used the same visual analogue scales, as the baseline breathing focus phase described above. Following this, participants completed a third set of mood rating scales but this time in relation to their mood during the worry phase,^4^ and completed ratings about their experience of engaging in worry for five minutes. Specifically, they were asked to indicate the percentage of time they spent worrying, how difficult they found it to worry, and how stressed they felt whilst worrying. The latter two items were each rated on a scale ranging from 0 (“not at all”) to 100 (“extremely”). Participants were also asked to retrospectively rate the percentage of time that their thought content was positive, negative or neutral.

### Manipulation check

To ensure that participants engaged in the designated mentation style, they completed two visual analogue scales at the end of the session as a manipulation check: one scale indicated the percentage of time they had engaged in verbal processing, and the other indicating the percentage of time they had engaged in imagery during the worry phase. Participants were included only if they scored above 55% on their designated mentation style and if this was at least 10% greater than the alternative style, as rated on the second visual analogue scale. Seventeen participants had to be excluded on this basis (2 participants indicated that they had not worried using verbal processing, and 15 indicated that they had not worried in imagery, despite being requested to do so) during the five-minute worry phase.^5^

### Description of cognitive intrusions

Finally, participants were asked to give a full description of each cognitive intrusion they had reported during the two breathing focus phases. Participants were reminded of their summary for each intrusion and asked to give a fuller description of what was going through their mind at the time of the tone, without reporting its valence. These descriptions were recorded onto a digital voice recorder and later rated for valence (positive, neutral or negative) by an assessor not informed of group allocation or from which phase the descriptions came (baseline or post-worry). In addition, a graduate psychologist rated a random subset of 25 percent of participants' expanded descriptions in order to provide inter-rater reliability, Kappa = .55, *p* < .001.

## Results

### Negative intrusions

Mean number of negative intrusions during the two breathing focus periods are presented in Table 2. A mixed-model ANOVA with within-subjects factors of time (baseline and post-worry) and rater (self-rating or assessor) and the between-subjects factor of group (verbal or imagery) revealed a significant effect of group, *F*(1, 57) = 8.29, *p* < .01, *f* = .4.^6^ There was no significant main effect of time, *F*(1, 57) = .77, *ns*, *f* = .05, or of rater, *F*(1, 57) = .20, *ns*, *f* = .07. However, there was a significant time by group interaction, *F*(1, 57) = 15.38, *p* < .001, *f* = .42, indicating that groups changed differently over time, with the verbal group showing a greater number of negative thought intrusions and the imagery group showing a reduced number of negative intrusions following the instructed worry period. This interaction remained significant when the percentage of negative cognitions experienced during the worry phase (as assessed by retrospective self-ratings) was included as a covariate, *F*(1, 57) = 6.74, *p* < .05, *f* = .34. Similarly, when self-reported time engaged in worry during the worry phase was used as a covariate, the time by group interaction again remained significant, *F*(1, 57) = 8.42, *p* < .05, *f* = .38. Thus the interaction effect was not accounted for by group differences in percentage of negative cognitions experienced during the worry phase, or self-reported time engaged in worry during the worry phase. All other effects were non-significant.

To further investigate the time by group interaction, post-hoc *t*-tests, using Hochberg's Improved Bonferroni Method (Hochberg, 1988), were conducted on number of negative intrusions at baseline and post-worry. There were no significant differences in the number of negative intrusions reported by the verbal and imagery group or rated by the assessor before the instructed worry period. In contrast, following the worry period, the imagery group reported significantly fewer intrusions (see Table 2 for details).

Furthermore, paired-samples *t*-tests indicated that the verbal group displayed a significant increase in the number of negative intrusions from baseline to post-worry whereas the imagery group displayed a significant decrease from baseline to post-worry (see Table 2).

### Mood

To investigate the impact of the two conditions on participants' self-reported anxiety, a mixed-model ANOVA was performed with a within-subjects factor of time (baseline, worry period and post-worry) and a between-subjects factor of group (verbal processing or imagery). This revealed no significant interaction between time and group, *F*(1, 52) = 1.53, *ns*, *f* = .18, indicating that the groups did not differ in their levels of self-reported anxiety at any point during the experiment. There was, however, a significant main effect of time, *F*(2, 51) = 47.34, *p* < .001, *f* = 1.36. A series of paired-samples *t*-tests revealed that anxiety significantly increased during the worry period, *t*(52) = −8.14, *p* < .001, *d* = 2.26, and then significantly reduced after the second breathing focus phase, *t*(52) = −8.75, *p* < .001, *d* = 2.44, to approximately baseline level, *t*(58) = −1.81, *ns*, *d* = .48.

Mixed-model ANOVAs conducted on depression ratings yielded the same pattern of results, while those on happiness ratings showed the analogous (converse) effects (Table 3).

### Additional analyses

Participants retrospectively rated what proportion of their cognitions during the five-minute worry phase was positive, neutral or negative. In addition, they retrospectively rated the percentage of time they engaged in worry during the worry period, the percentage of time they engaged in the assigned mentation style (verbal thought or imagery), the difficulty with which they worried, and the level of stress experienced during the worry period. There were no group differences on these ratings except for the proportion of negative cognitions during the worry period, which was slightly higher in the verbal group. Table 4 presents these results.

Participants' descriptions of their worry topic were recorded by the experimenter and rated by an assessor for degree of negativity using a three point scale: low, moderate, or high. A graduate psychologist rated a random subset of 25 percent of these worry topics in order to provide inter-rater reliability, Kappa = .44, *p* < .05. Also, the domain of each worry topic was classified into one of four categories: work/study worries, social/relationship worries, financial worries, and physical worries, and a random sample of 25% of these were also rated by a graduate psychologist rated in order to provide inter-rater reliability, Kappa = .78, *p* < .001. There were no significant group differences in the degree of negativity of worry topics, *P* = .52, *ns* (Fisher's exact probability test), with both reporting a predominance of moderate worries (24 out of 30 in the verbal group and 27 out of 30 in the imagery group). In addition, there were no group differences in the relative frequency of the domain of the worry topics, *P* = 5.41, *ns* (Fisher's exact probability test), with both reporting a preponderance of work/study related worries and social/relationship worries.

## Discussion

Instructions to worry verbally (as is usual in worry) resulted in an increase in reported negative thought intrusions during the subsequent breathing-focused phase, while instructions to worry using imagery led to a decrease. These results were not confined to participants' own subjective ratings, but held when descriptions of thought intrusions were rated by an assessor not informed of group allocation.

Participants in the verbal group also reported a higher proportion of negative cognitions during the five-minute worry phase itself. However, effects on post-worry intrusions remained significant when either the time rated as spent worrying during the instructed worry phase or the proportion of negative thoughts experienced during the worry phase were covaried out. Furthermore, differences in the number of negative intrusions post-worry could not be accounted for by trait worry level, mood, number of negative intrusions before the worry phase, relative negativity of worry topic or domain of worry topic experienced during the worry phase.

The current study also explored the impact of engaging in imagery of a current worry topic on mood. There was no differential impact of mentation style on mood: self-reported anxiety and depressed mood increased while happiness decreased during the worry period in both groups. These findings differ from some previous studies. Butler et al. (1995) demonstrated that verbally worrying about a distressing video was associated with a greater decrease in self-reported anxiety compared to imagery. Nelson and Harvey (2002) did not obtain pre-worry measures but found that imagining giving a speech was associated with greater negative affect than worrying about it. However, unlike the current study, these studies introduced a stressor prior to the worry phase, and in the case of Butler et al. this in itself raised their anxiety. Participants were then asked to worry about the stressor, whereas in the current study, participants were instructed to think about their own currently most worrisome topic. Participants' idiosyncratic worries would have been thought about repeatedly prior to the study, and this may have had a different impact on mood than being asked to think about a novel negative topic or stressor. Alternatively, the lack of differences in mood between the groups may be due to retrospective ratings, which may be insensitive to subtle changes in mood. Furthermore, not all the previous literature shows consistent findings. Behar and Borkovec (2005) found that worrying in imagery was not associated with any change in self-reported distress (albeit this may be different to anxiety) whereas verbal worry was associated with an increase. Further research is therefore needed before firm conclusions can be drawn regarding the impact of mentation style on affect.

It should be noted that a number of participants had to be excluded from the imagery condition (*n* = 15) on the basis of not being able to engage in imagery about their worry topic, whereas only two participants were excluded from the verbal condition for not being able to engage in verbal worry. While it was important to exclude these participants from the analyses because they had not followed the instructions sufficiently to test the hypothesis, the results remained unchanged when they were included in the analysis. However, given the higher rates of exclusion from the imagery group, engaging in imagery about a current worry topic is clearly a difficult task for high worriers, highlighting the need for further refinement of the imagery training in order to help high worriers and individuals with GAD worry less. Future studies may focus on enhancing the training phase, which may involve a greater number of practice trials, including some practice trials of less pertinent worry topics, prior to the five-minute experimental worry phase.

It has already been established that worry is primarily a verbal process (e.g. Borkovec & Inz, 1990), and therefore it is likely that the verbal condition in the current study is similar to the usual phenomenology of worry. In keeping with this, the increase in negative intrusions after a period of verbal worry is consistent with previous studies (Borkovec et al., 1983; Ruscio & Borkovec, 2004). However, the current study is the first to directly compare a period of verbal worry with a period of imaginal worry whilst utilizing participants' real-life worry topics. The finding that only participants worrying in verbal form displayed an increase in negative intrusions is consistent with the hypothesis that the verbal nature of worry may be one of the key factors in its maintenance and uncontrollability (Sibrava & Borkovec, 2006).

This is the first study to demonstrate that worrying using imagery is associated with a *decrease* in negative intrusions. There are a number of reasons why this might be so. First, verbal worry may be problematic in terms of its highly abstract content. Many worrisome thoughts are of the *“what if…?”* type, relating to uncertain outcomes (Stöber & Borkovec, 2002), and thus it is plausible that they lack a specific context as well as being rather fragmented. The abstract and fragmented nature of the worrisome thoughts may allow the worrier to jump from one topic to another, and reach catastrophic outcomes which exacerbate further worry intrusions. In contrast, generating imagery may be a more helpful process. Imagery appears to have strong links with memory; for example, Dewhurst and Conway (1994) suggest that knowledge stored in long-term memory needs to be accessed and searched in order to generate images. Thus generating imagery may draw on autobiographical memories and the individual's knowledge of the world, facilitating a more specific and concrete mental representation of the worry topic. It is likely that this study may have been the first time participants had considered their worry topics using imagery, and the image generated may have acted as an on-line test of their negative catastrophic ideas, leading to changes in appraisals of the situation, either because the image did not correspond with their previous catastrophic ideas, or because the catastrophic image generated seemed unrealistic in light of their knowledge of the real world. Imagery may, therefore, provide a way of facilitating helpful reappraisals of the worry topic. This is consistent with Foa and Kozak's (1986) emotional processing theory, which postulates that the presence of corrective information during exposure to the fear stimulus is necessary for fear extinction to occur. It may be that imagery per se is not necessary for this process to take place, and that other mentation styles that similarly facilitate reappraisals of the worry topic may be equally effective in reducing further negative thought intrusions. Future research needs to investigate this.

The current results therefore provide some support for the hypothesis that the verbal nature of worry contributes to its maintenance (Sibrava & Borkovec, 2006), and that imagery may be helpful in reducing negative thought intrusions. The results of this study also suggest that the way in which negative information is represented during worry is amenable to change, with potential beneficial consequences for the modification of subsequent negative intrusions. In future research an imagery training paradigm based on the one used here could be used with clients with GAD to investigate whether training them to process worrisome topics in this way is beneficial, thus supporting the inclusion of imagery in cognitive behavioural treatments for this disabling condition.

## Figures and Tables

**Table 1:** Mean (SD) participant characteristics.

	Verbal	Imagery	*t* (58)
Age	26.83 (13.44)	25.34 (8.69)	.01
PSWQ	67.23 (5.58)	65.21 (5.43)	1.18
STAI-S	48.50 (10.59)	45.31 (8.03)	1.43
STAI-T	54.43 (7.00)	51.38 (5.92)	1.81
HADS-Anxiety	11.63 (3.42)	11.03 (3.27)	.78
HADS-Depression	5.07 (3.68)	4.28 (2.77)	.96

Note. PSWQ: Penn State Worry Questionnaire; STAI-S: Spielberger State-Trait Anxiety Inventory – State; STAI-T: Spielberger State-Trait Anxiety Inventory – Trait; HADS: Hospital Anxiety and Depression Scale. All tests were non-significant.

**Table 2:** Mean (SD) negative intrusions (out of 12) during breathing focus phase.

	Verbal group	Imagery group
Before worry phase		
Participant-rated	2.50 (1.85) _a_	2.00 (1.64) _a_
Assessor-rated	2.20 (1.70) _a_	2.31 (1.84) _a_

After worry phase		
Participant-rated	3.30 (2.26) _b_	1.33 (1.21) _c_
Assessor-rated	3.21 (1.84) _b_	1.55 (1.21) _c_
N	30	30

Note. Means with different subscripts differ significantly at at least the *p* < .05 level.

**Table 3:** Mean (SD) mood ratings before, during and after the worry phase.

	Verbal group			Imagery group		
	Baseline	Worry phase	Post-worry	Baseline	Worry phase	Post-worry
Anxious	40.58 (24.62)_a_	71.61 (17.40) _b_	50.08 (23.64) _a_	37.89 (18.95) _a_	65.18 (23.73) _b_	41.96 (19.34) _a_
Depressed	23.85 (19.05)_c_	46.62 (26.88) _d_	31.81 (23.14) _c_	27.29 (18.76) _c_	47.32 (27.77) _d_	29.29 (18.94) _c_
Happy	39.81 (14.88) _e_	73.15 (10.64)_f_	45.77 (13.24)_e_	40.68 (10.95) _e_	66.79 (21.09) _f_	42.86 (12.05) _e_

Note: Happiness ratings have been reverse scored. Means with different subscripts across mood states are significantly different at at least the *p* < .05 level.

**Table 4:** Retrospective ratings of the worry phase.

		Verbal	Imagery	t (58)	p
Cognitions during worry	Positive	12.67 (14.61)	19.67 (24.88)	−1.33	N.S.
	Neutral	20.67 (14.55)	24.73 (20.70)	-.88	N.S.
	Negative	68.18 (17.24)	53.52 (29.27)	2.74	< .05
Time spent worrying		79.93 (17.82)	72.50 (22.47)	1.42	N.S.
Time engaged in assigned mentation style		76.50 (15.98)	74.80 (12.44)	.46	N.S.
Difficulty worrying		32.82 (29.59)	26.30 (31.06)	.834	N.S.
Stress during worry		67.33 (21.04)	59.00 (26.21)	1.36	N.S.

## Footnotes

2 Prior research with college samples (Molina & Borkovec, 1994) found a PSWQ score of 56 to fall one standard deviation below the mean of individuals diagnosed with GAD by the Anxiety Disorders Interview Schedule for DSM-IV (ADIS-IV; Brown, Di Nardo, & Barlow, 1994).

3 These topics differed from the topics used in the imagery training phase. This was because it was found during piloting that asking participants in the verbal group to generate their own topics resulted in a large proportion of imagery, whereas abstract topics did not, more in keeping with worry.

4 Ratings were made retrospectively to avoid diluting the effects of the worry period on the subsequent breathing focus phase.

5 Excluded participants' scores on all standardised measures were not significantly different to the rest of the sample. When excluded participants' data were included in the overall analyses, the patterns of results remained unchanged.

6 A Cohen's *f* of .10 is considered a small effect, .25 medium, and .40 a large effect size.
